# Homology Modeling and Molecular Dynamics-Driven Search for Natural Inhibitors That Universally Target Receptor-Binding Domain of Spike Glycoprotein in SARS-CoV-2 Variants

**DOI:** 10.3390/molecules27217336

**Published:** 2022-10-28

**Authors:** Olha Ovchynnykova, Karina Kapusta, Natalia Sizochenko, Kostyantyn M. Sukhyy, Wojciech Kolodziejczyk, Glake A. Hill, Julia Saloni

**Affiliations:** 1 Department of Fuel, Polymer, and Polygraphic Materials Technologies, Ukrainian State University of Chemical Technology, 49005 Dnipro, Ukraine; 2 Department of Chemistry and Physics, Tougaloo College, Tougaloo, MS 39174, USA; 3 The Ronin Institute for Independent Scholarship, Montclair, NJ 07043, USA; 4 Interdisciplinary Center for Nanotoxicity, Department of Chemistry, Physics and Atmospheric Sciences, Jackson State University, Jackson, MS 39217, USA

**Keywords:** COVID-19, SARS-CoV-2 mutations, receptor-binding domain, molecular docking, molecular mechanics, molecular dynamics, homology modeling, natural compounds, disinfectors

## Abstract

The rapid spread of SARS-CoV-2 required immediate actions to control the transmission of the virus and minimize its impact on humanity. An extensive mutation rate of this viral genome contributes to the virus’ ability to quickly adapt to environmental changes, impacts transmissibility and antigenicity, and may facilitate immune escape. Therefore, it is of great interest for researchers working in vaccine development and drug design to consider the impact of mutations on virus-drug interactions. Here, we propose a multitarget drug discovery pipeline for identifying potential drug candidates which can efficiently inhibit the Receptor Binding Domain (RBD) of spike glycoproteins from different variants of SARS-CoV-2. Eight homology models of RBDs for selected variants were created and validated using reference crystal structures. We then investigated interactions between host receptor ACE2 and RBDs from nine variants of SARS-CoV-2. It led us to conclude that efficient multi-variant targeting drugs should be capable of blocking residues Q(R)493 and N487 in RBDs. Using methods of molecular docking, molecular mechanics, and molecular dynamics, we identified three lead compounds (hesperidin, narirutin, and neohesperidin) suitable for multitarget SARS-CoV-2 inhibition. These compounds are flavanone glycosides found in citrus fruits – an active ingredient of Traditional Chinese Medicines. The developed pipeline can be further used to (1) model mutants for which crystal structures are not yet available and (2) scan a more extensive library of compounds against other mutated viral proteins.

## 1. Introduction

**Figure 1:**
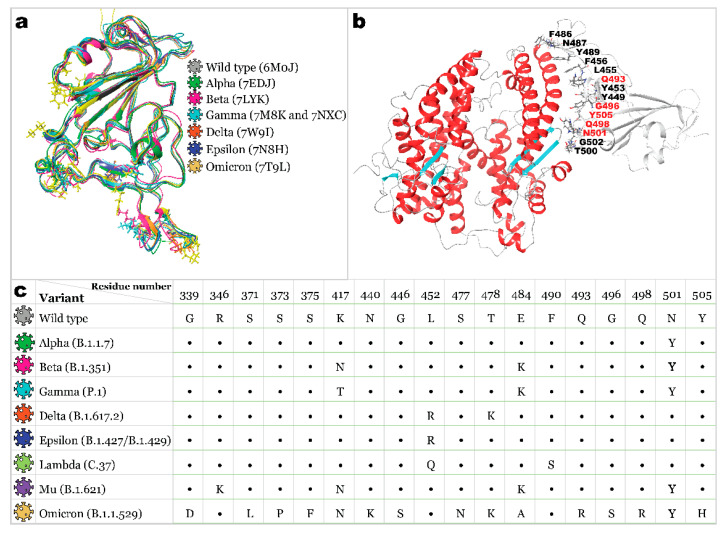
Structures of the SARS-CoV-2 S protein RBD: (**a**)—superposition of available RBD 3D structures with the ball-and-stick representation of mutated residues (ribbons and residues colored based on variant); (**b**)—wild-type RBD bound to ACE2 (PDB ID: 6M0J) with RBD residues reported to interact with ACE2 being marked, and mutating residues’ labels being colored in red; (**c**)—list of occurred mutations for variants studied here.

Coronavirus Infectious Disease-19 (COVID-19) caused by Severe Acute Respiratory Syndrome Coronavirus 2 (SARS-CoV-2) has become a primary foe for humanity as the virus has been rapidly spreading over the globe since 2019, causing a high sickness rate, deaths, and triggering a global economic crisis. For the last 2 years, the focal point of interest of the research community has narrowed down to the development of vaccines and medicines to stop an ongoing pandemic. At this stage, many therapeutics were tested and recommended for treating COVID-19 [1,2,3]. However, the only drug currently authorized by the Food and Drug Administration (FDA) is the oral antiviral Paxlovid [4]. Several immunization agents have already been released, including replication-defective viral vector vaccines, inactivated pathogen vaccines, protein subunit vaccines, virus-like vaccines, and novel mRNA vaccines [5]. However, the documented research lacks evidence that already-developed therapeutics will remain effective against every existing and emerging variant of SARS-CoV-2 [6,7]. The binding of the human angiotensin-converting enzyme 2 (ACE2) receptor with the receptor-binding domain (RBD) of the SARS-CoV-2 spike protein is attributed to be a major determinant of viral infectivity and spread [8,9]. Therefore, RBD is a promising target for vaccine development and drug discovery. With an extensive mutation rate of the virus (the receptor-binding domain is estimated to have about 24 substitutions per year [10]), scientists need to be prepared for the emergence of new mutated strains with mutations that increase evasion of the antibody response (Figure 1).

The viral variant with the D614G mutation in spike glycoprotein emerged in early 2020 and became the first indication of SARS-CoV-2 genetic evolutionary selection [11]. This mutation is responsible for easier transmission, an increased number of spike proteins per virion, and a more significant S1/S2 cleavage rate [12,13]. Further, in December 2020, the Alpha variant appeared in the UK, carrying mutations such as Δ69/70, Δ144, N501Y, and P681H. An important N501Y mutation in RBD increased the affinity of viral spikes to the ACE2 host cell protein [14,15]. Moreover, Beta, Gamma, Mu, and the newest Omicron variants’ RBDs also carry this mutation. Next, Beta and Gamma variants originating from South Africa and Brazil carry additional E484K and K417N/T mutations. K417N/T mutation, presumably, reduced the spike protein’s binding affinity to ACE2 [16,17,18]. However, it did not make these variants less transmittable since all three mutations had compensatory effects on ACE2 binding [19]. These two variants raised concerns due to increased resistance to antibody neutralization, facilitating immune escape [20,21,22,23]. The Delta variant (B.1.617.2), which emerged from India, outperformed all previously prevalent variants and became a dominant strain worldwide [24]. Carrying only two mutations in RBD (L452R and T478K), it became highly transmittable and more resistant to neutralizing antibodies [24]. Other emerging variants did not gain widespread attention until the Omicron variant was first reported to the World Health Organization (WHO) by South Africa on 24 November 2021. It showed a record-breaking number of mutations with 15 substitutions within the RBD [25,26,27], which resulted in viral escape from most existing SARS-CoV-2 neutralizing antibodies [28,29,30,31,32]. According to the subsampling of globally circulating sequences created by NextStrain, the spread of most variants was diminished by December 2021. Delta and Omicron variants are still of primary concern worldwide as of January 2022 [10].

To date, a record-breaking number of research articles have been devoted to computational drug design for COVID-19. As a result, numerous research teams have produced low-quality computations based solely on molecular docking, which are not accurate enough to reasonably serve as a practical guide for any experimental research. Nonetheless, some exceptional works worth noticing showcase sophisticated state-of-the-art computations, such as accurate molecular mechanics and molecular dynamics techniques. For instance, in [33], authors used multiple 500 ns molecular dynamics simulations, utilizing the Schrödinger Software Package to assess the stability and interfacial interactions of SARS-CoV-2 and its predecessor SARS-CoV RCBs with host receptor. The 200 ns Molecular Dynamics simulations performed by researchers in [34] revealed that the SARS-CoV-2 spike glycoprotein completely inactivates at a temperature of 50 °C. Various computational technics were used in [35] to evaluate the conformational accessibility and binding affinity of a wild type of SARS-CoV-2 spike glycoprotein to ACE2. Overall, to produce reliable computational results, researchers should not rely on only one method but rather a comprehensive modeling pipeline that utilizes various approaches to ensure the accuracy of calculations.

With the prevailing problem of a high mutation rate, developing inhibitors capable of binding to viral proteins regardless of mutations is of significant importance. In this case, computational approaches such as homology modeling may become a valuable tool at a time when the crystal structure of new mutants is not yet available [36]. Such approaches, if accurate enough, may serve as a guide for experimental testing and thus have the potential to accelerate a drug discovery pipeline.

This work presents a computational modeling pipeline designed to identify natural wide-range inhibitors of interactions between SARS-CoV-2 spike glycoprotein and ACE2 host protein. It reports eight validated using experimental data [37] homology models of RBDs of different SARS-CoV-2 variants. Developed models and reference structures are further utilized to elucidate interactions with a host receptor, aiming to determine which critical residues for receptor recognition can be used as target residues for potential drugs. Finally, the benchmark model is created for multitarget scanning the potential inhibitors’ library against various strains of the SARS-CoV-2 receptor binding domain.

## 2. Results and Discussion

### 2.1. Receptor Binding Domain Mutations and Homology Models of SARS-CoV-2 Variants

Homology models for receptor binding domains of Alpha (B.1.1.7), Beta (B.1.351), Gamma (P.1), Delta (B.1.617.2), Epsilon (B.1.427/B.1.429), Lambda (C.37), Mu (B.1.621), and Omicron (B.1.1.529) variants were built using wild type RBD (PDB ID: 6M0J) as a template. All eight models were subjected to 200 ns molecular dynamics simulation with further trajectory clustering to ensure a better quality of structures (Figure 1).

**Figure 2:**
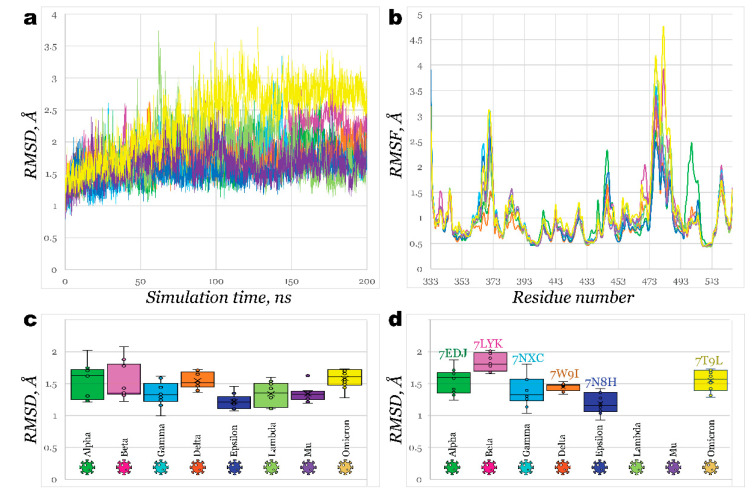
Homology models of SARS-CoV-2 variants’ RBDs: (**a**)—RMSD of generated homology models for 200 ns molecular dynamics simulation; (**b**)—RMSF of generated homology models for 200 ns molecular dynamics simulation; (**c**)—Box and whiskers graph for 10 clusters of each generated homology model, derived from MD simulation, representing its RMSD aligned on Wild type RBD’s structure (PDB ID: 6M0J); (**d**)—Box and whiskers graph for 10 clusters of generated homology models, representing each variant’s RMSD aligned on corresponding crystallographic structures.

For most structures, RMSD plots (Figure 2a) illustrated similar flexibility of models. The most significant fluctuations were observed for Lambda and Omicron (deviating slightly above 2.5 Å), while deviation for the rest of the models has not exceeded 2 Å. Delta and Epsilon RBDs were the most stable; both carry the L452R mutation. Analysis of root mean square fluctuations (Figure 2b) revealed a similar pattern of residue flexibility. The most considerable fluctuations were noticed near the region between Y473 and C488, as is the most evidential in Omicron, Beta, Gamma, Lambda, and Mu variants. Interestingly, in all these variants, except for Lambda, mutation of glutamic acid (E484) had occurred, substituting it with lysine or alanine (in the case of Omicron). E484K mutation, or so-called escape mutation, is responsible for reduced antibody affinity, thus immune escape. The structure of Alpha RBD significantly deviated from the common pattern with considerably more significant fluctuations near T500 at 2.5 Å.

**Figure 3:**
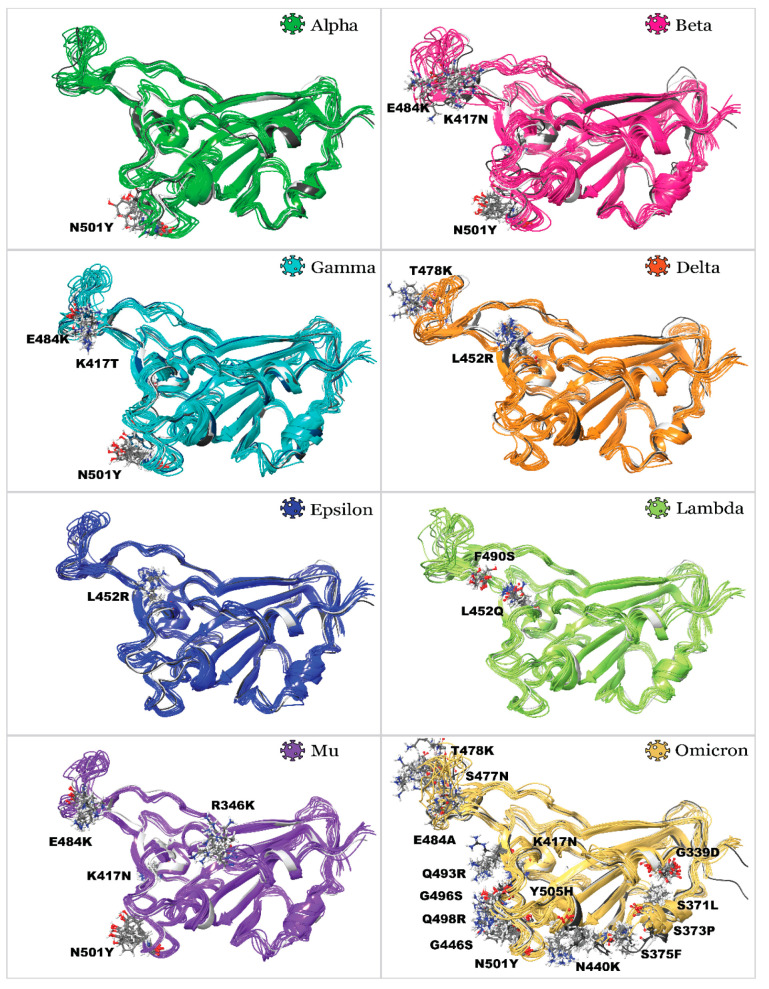
Superposition of 3D structures, illustrating 10 clusters of developed homology models of SARS-CoV-2 variants’ RBDs aligned on Wild type variant and reference PDB structures: mutated residues are labeled.

After analyzing molecular dynamics simulations, trajectory clustering was performed, generating the top 10 most populated clusters for each homology model. Comparison of RMSD of each cluster fit on Wild type variant structure is illustrated in Figure 2c as a box and whiskers plot. One can see that clusters for Epsilon variant RBD were the closest in structure to the Wild type, followed by Mu and Gamma variants. Meanwhile, RBDs of Beta, Omicron, Alpha, and Delta showed the least similarity to a Wild type structure. Developed homology models were also compared with existing PDB structures (Figure 2d). Generally, all models can be built effectively (Figure 3) with RMSD not exceeding 2 Å. The highest deviations were observed for the Beta variant model, and the lowest ones were detected for Epsilon. This study used the following reference structures (PDB IDs): 2AJF, 6M0J, 7EDJ, 7LYK, 7M8K (7NXC), 7W9I, 7N8H, and 7T9L. As of July 2022, no reference structures were available for variants’ Lambda and Mu RBDs.; therefore, we used homology models. It should be noted that we initially used the 7M8K structure of the Gamma variant’s RBD; however, this structure appeared considerably unstable during all further molecular dynamics simulations and showed unrealistically low binding towards ACE2, so this structure was substituted by PDB ID 7NXC.

### 2.2. Interactions of RBDs with ACE2

Understanding how RBD interacts with ACE2 can guide a search for the most suitable inhibitors. More specifically, knowing how conserved amino acid residues bind ACE2 despite the mutations, one can design a ligand that blocks specifically this region of RBD. To learn more details, RBD structures of SARS-CoV (PDB ID: 2AJF), Wild type of SARS-CoV-2 (PDB ID: 6M0J), Alpha (PDB ID: 7EDJ), Beta (PDB ID: 7LYK), Gamma (PDB ID: 7NXC), Delta (PDB ID: 7W9I), Epsilon (PDB ID: 7N8H), Omicron (PDB ID: 7T9L), Lambda and Mu (homology models, developed in this work) were combined with ACE2 protein (PDB IDs: 2AJF for SARS-CoV and 6M0J for SARS-CoV-2) and subjected to 100 ns MD simulation. With identical initial geometry of ACE2 combined with different RBDs, it was possible to minimize bias.

**Figure 4:**
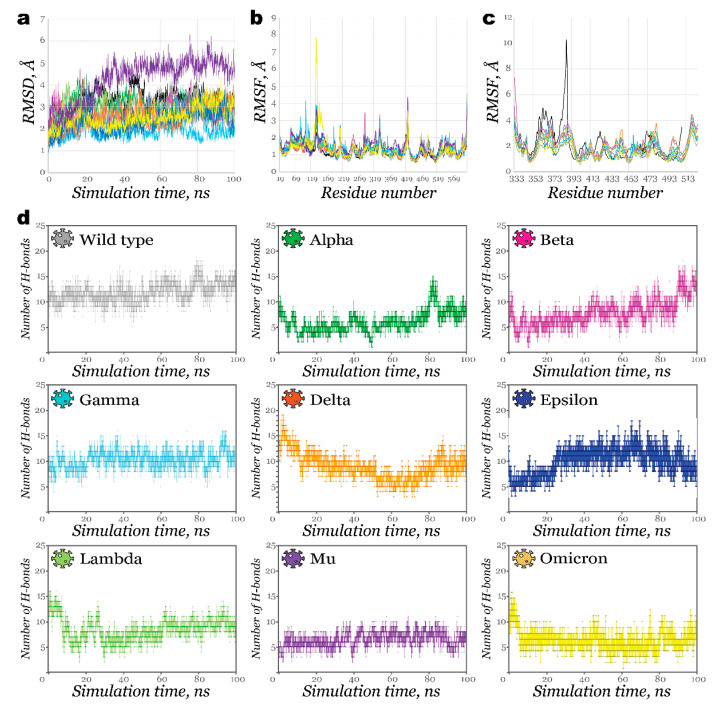
Results of 100 ns MD simulation for RBDACE2 complexes: (**a**)—RMSD of complexes (lines are colored based on the variant, black line illustrates RMSD of SARS-CoV RBDACE2 complex); (**b**)—RMSF of ACE2; (**c**)—RMSF of RBD; (**d**)—Number of hydrogen bonds retained during 100 ns MD simulation.

As expected, most structures showed large fluctuations on the RMSD graph from the beginning of simulations (Figure 4a), imitating the host-guest preparation step. Once complexes were stabilized, their deviations did not exceed 2 Å, except for the Delta RBD-ACE2 complex (less than 2.5 Å).

It took nearly 35 ns for complexes of Lambda and Mu RBD (homology models) with ACE2 to stabilize. This fact suggests that at least 100 ns simulations or even longer ones are required for proper investigation. As shown in Figure 4b, RMSF plots for ACE2 had similar fluctuations patterns in various regions for complexes of different variants. Only Omicron RBD bound to ACE2 caused its fluctuations to increase significantly near residues N134-E140. At the same time, the RMSF plot for RBD (Figure 4c) demonstrated more considerable flexibility for SARS-CoV. This fact agrees with available data for the stronger binding of SARS-CoV-2 with the host receptor compared to its predecessor [38].

The number of hydrogen bonds between these proteins as a function of time is presented in Figure 4d. During most simulation time, Alpha, Mu, and Omicron RBD complexes formed fewer than 10 H-bonds. Some affinity improvement towards the end of the simulation was observed for the Alpha variant. The complex with Lambda showed a significantly larger number of interactions at the beginning of the simulation, but this number declined as the simulation continued. Contrary to this, for the Beta variant, the number of H-bonds increased gradually, with the largest number observed after 90 ns. The strongest affinity was noticed in the case of the Gamma and Epsilon variant. The more significant number of H-bonds observed in the Wild typeACE2 complex is somewhat biased since the complex was initially more similar in geometry to the reference RBDACE2 complex. These results, excluding ones for a Wild type (for the above-mentioned reasons) and an Alpha variant, correlate with experimental findings obtained by Han et al. [37]. Authors measured binding affinities of the RBDs to ACE2 using a surface plasmon resonance assay to find out that RBDs from Alpha, Beta, and Gamma demonstrated enhanced affinities to the host protein, superior to RBDs of Omicron and Delta.

**Figure 5:**
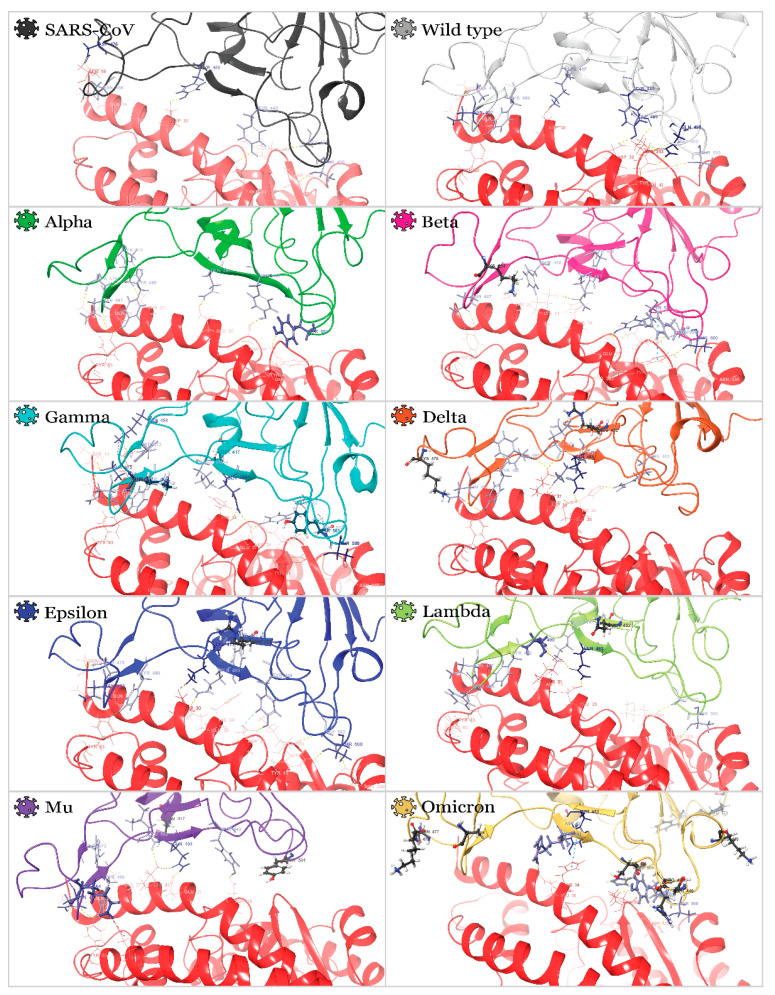
3D structures of RBDACE2 complexes are represented by the most populated cluster derived from MD simulations: ACE2 residues, which participate in interactions with RBD, are illustrated with wire representation and are colored from pink to dark red in order of increased number of interactions. RBD residues, which participate in interactions with ACE2, are illustrated by thin tube representation and are colored from light blue to dark blue in order of increased number of interactions. Mutated residues are illustrated with ball-and-stick representation.

Next, the outputs from molecular dynamics simulations were clustered to analyze which residues participate in interactions. The most populated cluster was used for interaction analysis (Table 1 and Figure 5).

It was found that the critical residues for almost all variants’ complexes are N487 and Q493. Many residues were earlier identified as essential for RBD’s association with ACE2 (Figure 1b)–although it must be noted that these two are the most conserved ones disregarding the mutations in the case of all nine studied variants. Thus, we hypothesize that inhibitors blocking these residues may be beneficial if targeting not just one variant but all of them simultaneously. N487 forms H-bonds with Y83 and Q24 (in some cases) of ACE2; Q493 (R493 in the case of the Omicron variant) forms H-bonds with either K31, H34, E35, or D38 (depending on the variant). Other important residues are K417, Y489, T500, G502, and Y505. All residues conform to those reported earlier in the literature [33]. Positively charged K417 formed H-bond and salt bridge with negatively charged D30 residue of ACE2. Residue 417 did not interact with ACE2 for Alpha, Beta, Gamma, Mu, and Omicron variants because of the mutation of positively charged lysine residue into an uncharged polar one (K417N or K417T mutations for all variants except for Alpha), which was confirmed by experimental investigation in [39]. 

Overall, the most significant number of interactions was observed in Gamma and Epsilon RBDs (as indicated in Table 1 and Figure 5). At the same time, the Lambda RBD had a relatively small number of interactions: only 5 H-bonds and one salt bridge were formed. Similarly, poor association with ACE2 was noticed for the Omicron variant. Interestingly, both the simulation of the retrieved from the PDB databank Omicron RBD (7T9L) and the simulation of the homology model developed here showed similar results.

### 2.3. Benchmark Models for Drug Binding

**Figure 6:**
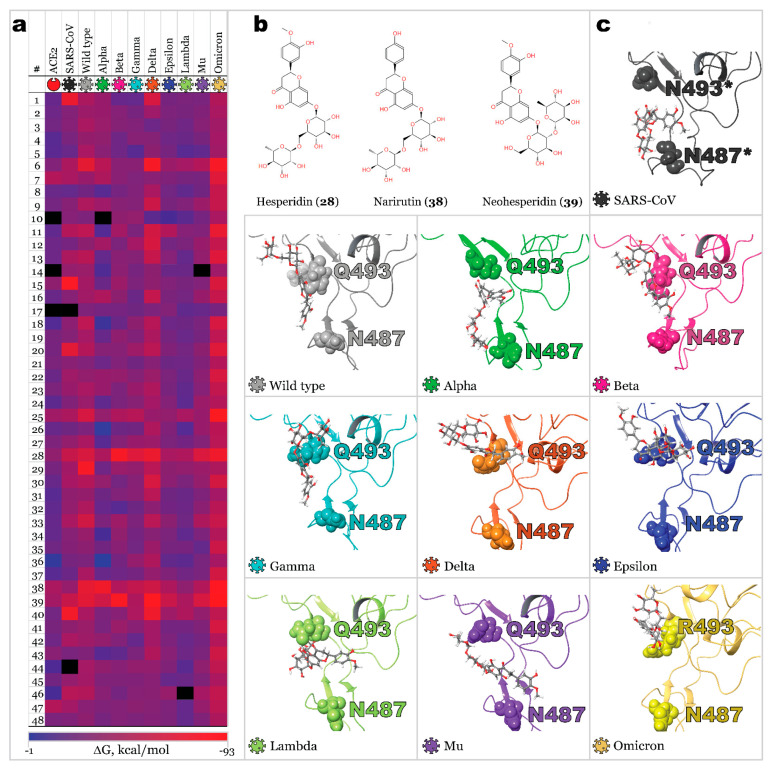
Results of MM-GBSA calculation: (**a**)—Free binding energies of ligand-protein complexes presented as heat maps; (**b**)—Active compounds with the highest averaged affinity towards most SARS-CoV-2 RBD variants; (**c**)—3D structures of RBDs complexed with compound **28** (residues of interest are illustrated with CPK representation). * original sequence numeration for SARS-CoV was changed in this figure according to the alignment of residues for easier comparison.

A small library of 48 active ingredients of some TCMs (Table 2) was selected and scanned against ACE2 and S-protein RBD of SARS-CoV and SARS-CoV-2 variants. This dataset of natural compounds was chosen purely for a benchmark purpose due to its evident efficacy in treating COVID-19, as stated in [40]. The ligand poses produced by molecular docking with the highest scoring modes were subjected to MM-GBSA calculation, enabling free binding energy evaluation and utilizing protein flexibility for all residues within 12 Å from a ligand. Calculated free binding energies are collected in Table S1 in the Supporting Information file. A heat map illustrating a binding affinity of ligands to human ACE2, RBD of SARS-CoV, and nine variants of SARS-CoV-2 is presented in Figure 6a. For most ligands, binding affinity with ACE2 was significantly lower than the affinity for viral spike protein. Moreover, for viral RBDs, a similar efficacy pattern was observed. Nonetheless, some ligands demonstrated high affinity to a single RBD and low affinity to all other RBDs. The highest averaged free binding energy was calculated for compounds **5**, **10**, **17**, **26**, and **36**, varying from −31.45 to −33.54 kcal/mol. 

The most efficient blockers (based on averaged free binding energy) are ligands **28** (hesperidin), **38** (narirutin), and **39** (neohesperidin) (Figure 6b), with free binding energies of −62.3, −59.28, and −66.62 kcal/mol, respectively. These findings are in agreement with existing experimental data [41,42] and reported computational results [43,44,45]. It is worth noting that these best-scoring compounds are present in citrus essential oils and orange juice. Analysis of binding modes revealed that these compounds bind inside the proposed region (Figure 6c); however, the binding pattern for different variants is not the same as for RBD-**28** (RBD-hesperidin) complexes. Ligands bound along RBD-ACE2 binding surface direction in Wild type, Beta, Gamma, Delta, Epsilon, and Omicron RBD-hesperidin complexes. It caused the blocking of amino acid residue Q493 (R493 in the case of Omicron). Alpha variant’s complex had ligand dislocated closer towards N487. A similar binding mode was detected for Lambda variants’ complex, with hesperidin being associated perpendicularly to the RBD-ACE2 binding surface line, binding Q493. SARS-CoV had a similar pattern to the previous one, with the ligand dislocated closer to N487.

**Figure 7:**
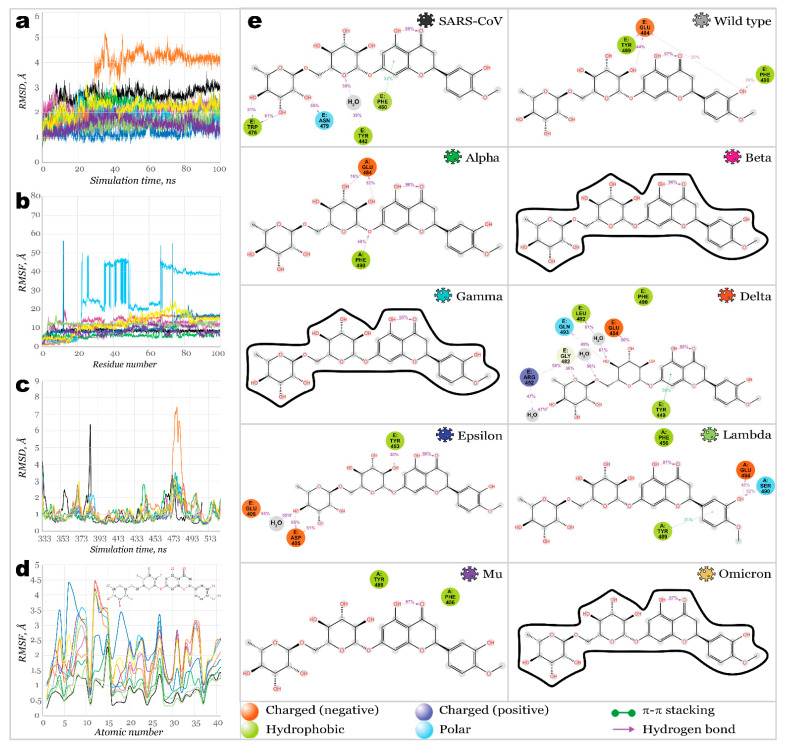
100 ns MD simulations results for RBD**28** (hesperidin) complexes: (**a**)—RMSD of protein’s *Ca*; (**b**)—RMSF of protein structures; (**c**)—RMSD of ligand fit on protein; (**d**)—RMSF of ligand’s structures; (**e**)—2D interactions diagram for hesperidin and RBDs.

Once these three potential multitarget inhibitors were identified using a heat map, their complexes with different RBDs were used to perform a 100 ns molecular dynamics simulation to evaluate their binding stability more accurately. Figure 7 illustrates proteins and ligands RMSD and RMSF plots resulting from 100 ns MD simulation for complexes of RBD-hesperidin (**28**).

Proteins of all variants were stabilized with RMSD fluctuations not exceeding 1.5 Å. The only exception was the complex with Delta RBD, with an observed significant deviation, stabilized after 50 ns of simulation. It may indicate a change in the protein’s conformation upon binding a ligand. The root mean square fluctuation within RBDs was significantly low, with the highest peaks for most SARS-CoV-2 variants below 3.6 Å. Only the Delta variant’s RBD showed a high fluctuation over 7 Å near residues S477-P479. Complex with SARS-CoV RBS demonstrated high fluctuations of 6.3 Å for the residue N388 (N375, according to the native sequence of SARS-CoV RBS), in the same way as it was for its complex with ACE2. Ligands fit on protein RMSD revealed extremely high deviation for the hesperidin complex with Gamma variants’ RBD, indicating that ligand was significantly departed from the original location. Ligand bound to Beta, Mu, and Omicron RBDs also showed high deviations.

Stepped change in ligand’s position fit can be observed for Epsilon RBD: stabilizing during the first 65 ns of a simulation time and further changing its position and stabilizing again after 85 ns. Overall, hesperidin was fixed the strongest inside of binding pockets of SARS-CoV, Wild type, Alpha, Delta, Epsilon, and Lambda SARS-CoV-2 variants’ RBDs. A similar binding pattern can be seen in cases of Wild type, Alpha, and Delta RBDs complexed with hesperidin. Hydroxyl groups of the glucose ring served here as donors of hydrogen bonds to E484 (ranging from 44% to 76% of a simulation time). At the same time, one more H-bond was formed between hydrophobic F490 and the hydroxyl group of phenyl ring (Wild type, 36% of a simulation time) or linkage oxygen (Alpha, 48% of a simulation time). RBD residues, which interacted with hesperidin, were similar in the case of Lambda RBD; however, only the phenyl ring of a ligand participated in interactions, forming π-π stacking with Y489 and H-bonds with E484 and S490. Considering the importance of E484 in promoting the binding of a ligand, one can assume that mutation of this negatively charged amino acid into positively charged lysine (or neutral alanine) should result in reduced binding affinity. Indeed, no contacts between hesperidin and the protein were observed in all variants carrying the mutation E484K or E484A.

**Figure 8:**
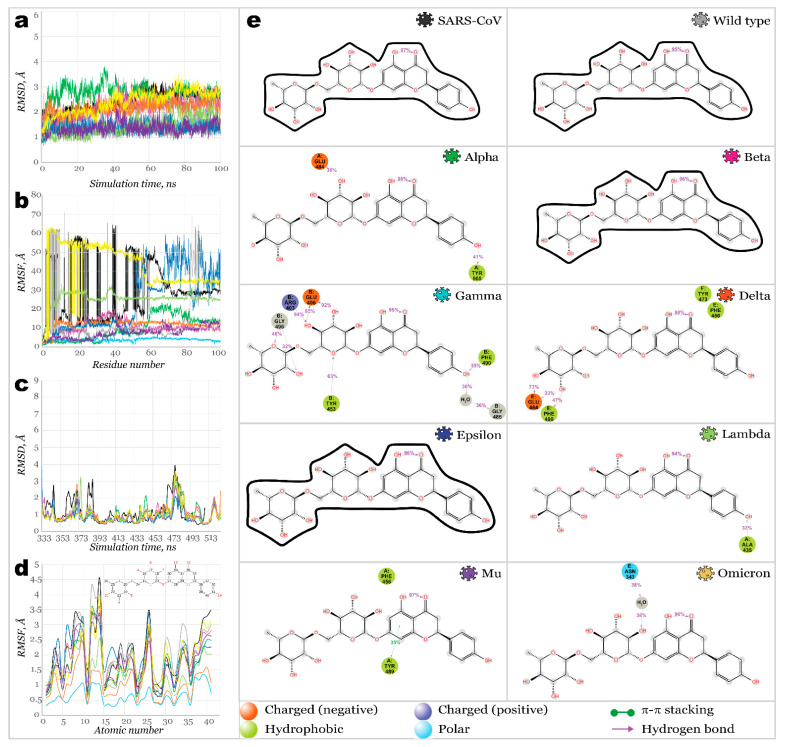
100 ns MD simulations results for RBD**38** (narirutin) complexes: (**a**)—RMSD of protein’s *Ca*; (**b**)—RMSF of protein structures; (**c**)—RMSD of ligand fit on protein; (**d**)—RMSF of ligand’s structures; (**e**)—2D interactions diagram for narirutin and RBDs.

Narirutin (**38**) showed a weaker affinity toward SARS-CoV and SARS-CoV-2 variants (Figure 8). As shown in Figure 8a, protein RMSD was stabilized, not exceeding 2 Å. However, the ligand’s RMSD fit on protein revealed substantial changes in the ligand’s position for most complexes. Ligands RMSF showed large fluctuations for most complexes except for Gamma and Delta. Narirutin formed the most stable complexes with Alpha, Gamma, and Delta RBDs. Sever H-bonds and a water bridge have strongly stabilized the Gamma variant’s RBD binding pocket. Next, H-bonding with E484 stabilized Narirutin complexes with Alpha and Delta. However, in the case of an Alpha RBD, interactions with E484 remained shorter within the simulation time. Meanwhile, the Gamma variant’s RBD carried an E484K mutation, making it impossible to act as an H-bonds acceptor. Thus, instead of E484, this role was played by E406. Three other residues served as H-bond donors for the ligand, maintaining contacts for 32–94% of the simulation time. During 100 ns simulation time, narirutin was kept the strongest inside Gamma and Delta RBD’s binding pockets. Relatively weak affinity was detected for the Lambda, Mu, and Omicron RBDs, and no interactions were observed for complexes with SARS-CoV, Wild type, Beta, and Epsilon SARS-CoV-2.

**Figure 9:**
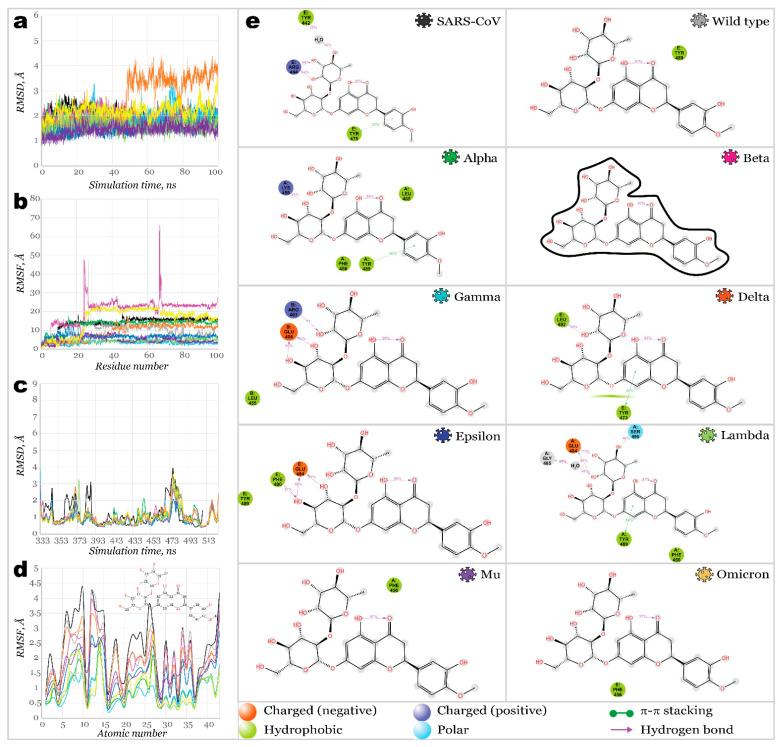
One hundred nanoseconds of MD simulations results for RBD-**39** (neohesperidin) complexes: (**a**)—RMSD of protein’s *Ca*; (**b**)—RMSF of protein structures; (**c**)—RMSD of ligand fit on protein; (**d**)—RMSF of ligand’s structures; (**e**)—2D interactions diagram for neohesperidin and RBDs.

Neohesperidin (**39**) appeared to be the most potent inhibitor among the ones discussed in this work. It bound the majority of studied RBDs (Figure 9), except for Beta, Mu, and Omicron ones. Fluctuations on protein RMSD were not exceeding 1.5 Å, with only deviations slightly below 2 Å for the Gamma and Omicron variants. Considerably large deviations on ligands RMSD were only observed for complexes with Wild type, Beta, and Mu variants. Next, the ligand was stabilized only after 50 ns and 25 ns for Omicron and Delta variants, respectively. For Gamma, Delta, and Lambda complexes, H-bonds with E484 (E406 in the Gamma variant) were retained during 77–98% of the simulation time.

After scanning a limited selection of biologically active compounds, which can be found in some Traditional Chinese Medicines, we were able to find leads that possibly can inhibit several variants of SARS-CoV-2 spike glycoprotein RBD with similar efficacy. For instance, all three hit ligands showed a great affinity towards the Delta variant, and two of them tightly bound the Gamma variant’s RBD. All three hit ligands are components of citrus essential oils and can be found in orange juice.

## 3. Materials and Methods

The Schrodinger software package [46] was used for all the simulations in this work.

### 3.1. Protein Preparation

The structures of a SARS-CoV and SARS-CoV-2 Wild type, Alpha (B.1.1.7), Beta (B.1.351), Gamma (P.1), Delta (B.1.617.2), Epsilon (B.1.427/B.1.429), and Omicron (B.1.1.529) spike (S) glycoprotein’s receptor-binding domains were retrieved from the Protein Databank (PDB IDs: 2AJF, 6M0J, 7EDJ, 7LYK, 7M8K (7NXC), 7W9I, 7N8H, and 7T9L, respectively). Every target protein was prepared using Protein Preparation Wizard [47] implemented in the Schrodinger. The bond orders were assigned, and all hydrogen atoms were added after removing the original hydrogens. Protonation states were generated using Epik [48] for a pH of 7.0 ± 2.0, the hydrogen bond network was optimized, and restrained minimization of all atoms was carried out using OPLS3e force fields [49]. The superposition of RBDs is illustrated in Figure 1a. For the Gamma variant’s RBD, the structure 7M8K was used initially; however, as simulations were carried out, this structure was found to be not appropriate for this type of investigation (as described in Section 2); therefore, 7M8K was substituted by 7NXC.

### 3.2. Homology Modeling

As the literature suggests [23], the residues responsible for the binding of RBD with ACE2 include Tyr449, Tyr453, Leu455, Phe456, Phe486, Asn487, Tyr489, Gln493, Gly496, Gln498, Thr500, Asn501, Gly502, Tyr505 (Figure 1b). For investigated variants, the mutations in RBD occurred in residues Gly339, Arg346, Ser371, Ser373, Ser375, Lys417, Asn440, Gly446, Leu452, Ser477, Thr478, Glu484, Phe490, Gln493, Gly496, Gln498, Asn501, and Tyr505, with only five of them appearing to bind to the host cell directly. The highest number of these mutations (15 overall) were detected for the Omicron variant.

The chain E containing RBD was extracted from wild-type RBD bound to ACE2 (PDB ID: 6M0J) and then was used as a template for homology modeling of Alpha (B.1.1.7), Beta (B.1.351), Gamma (P.1), Delta (B.1.617.2), Epsilon (B.1.427/B.1.429), Lambda (C.37), Mu (B.1.621), and Omicron (B.1.1.529) variants’ RBDs. The list of mutations is illustrated in Figure 1c. Homology models were created using Schrodinger’s Homology Modeling module [46]. Secondary structure prediction was carried out using the ClustalW alignment method, which works best for structures with high sequential similarity [50]. Models were aligned and further built using an energy-based method. All homology models were further subjected to 200 ns molecular dynamics simulation as described below. The Desmond Trajectory clustering method was used with the backbone as an RMSD matrix to extract the most repeated structures from the trajectory. The most populated clusters were used for further calculations.

### 3.3. Ligand Preparation

A total of 48 active ingredients of Traditional Chinese Medicines (TCM) [40] were used. The list of compounds, the TCM type they are found in, and their PubChem identifiers can be found in Table 2 and Table S2 (Supporting Information). The structures were retrieved from the PubChem database (https://pubchem.ncbi.nlm.nih.gov/ (accessed on 1 January 2022)) and prepared using LigPrep. Ionization states were generated using Epik at pH 7.0 ± 2.0, and structures were minimized using the OPLS3e force field.

### 3.4. Molecular Docking and Molecular Mechanics

For RBDs of all variants, the grids for molecular docking were centered on the RBD-ACE2 binding interface (Figure 1c) with the following coordinates x: −35.0, y: 32.0, z: 0, with a length of 36 Å, and a scaling factor value that was set to 1. The ACE2 receptor grid was centered on x: −35.0, y: 32.0, z: 10, with a length of 36 Å. The size of an inner box was set by default as a 10 Å cube. The extra precision (XP) docking calculations were carried out using Glide [51]. An OPLS3e force field was used with flexible ligands and rigid protein structures. The scaling factor was set to 1. Post-docking minimization with strain correction terms was performed for each docking calculation. The top hits from each XP simulation were used to perform a molecular mechanics/generalized Born surface area (MM-GBSA) calculation implemented in the Prime module [52]. With the VSGB solvation model and OPLS3e force field, protein residues were set flexible for 12.0 Å distance for all ligands processed. The protein-ligand complexes were ranked based on their binding free energy.

### 3.5. Molecular Dynamics Simulation

Molecular dynamics simulations allows to estimate more accurately the thermodynamics and kinetics associated with drug–target recognition and binding when compared to molecular docking and molecular mechanics methods [53,54], as it was shown in our previous works. Molecular dynamics simulations were carried out using the Desmond module [55]. Structures were placed in orthorhombic boxes with minimized size and solvated with single point charge (SPC) water molecules. Simulation systems were neutralized with Cl^-^ or Na^+^ counterions. All systems were subjected to Desmond’s default eight-stage relaxation protocol that preceded the actual run. The OPLS3e forcefield was used for all calculations. With a 25 ps recording time step, homology models of RBDs were simulated within 200 ns. It was shown in our previous work that while 100 ns simulations were performed for all other complexes. The trajectory clustering method (described in the Section 3.2) was used to retrieve the most populated geometries for further calculations. Simulation Interaction Diagram and Simulation Event Analyses were used to evaluate root-mean-square deviations (RMSD), root-mean-square fluctuations (RMSF), and ligand-protein interactions.

## 4. Conclusions

In this work, we built homology models of spike glycoprotein receptor-binding domains from eight SARS-CoV-2 variants as binding targets for the library of potential inhibitors. Comparing homology models with existing PDB structures indicated the quality of the developed models and computational techniques used to build them. Thus, it provides the opportunity to accelerate in silico research targeting newly emerged variants once the sequence of viral proteins becomes available. Analysis of RBD’s complexes with host ACE2 receptor revealed residues Q493 and N487 being not only essential to its binding (as was already proven) but also the most conserved, disregarding the mutations in the case of all nine variants studied here. This information directed us to propose a hypothesis that inhibition near these residues may be beneficial if targeting not just one variant but all of them simultaneously. Next, solely for benchmarking purposes, a set of biologically active compounds was screened against SARS-CoV and SARS-CoV-2 variants’ RBDs. Three analyzed ligands demonstrated the strongest binding affinity: hesperidin, narirutin, and neohesperidin. Molecular dynamics simulations demonstrated stable protein-ligand interaction for these compounds with RBDs of several variants (mainly Delta). With a high probability of interacting in a specific region of RBD (between residues Q493 and N487), these ligands are assumed to prevent interaction with RBD SARS-CoV-2. Interestingly, all three hit compounds can be found in citric essential oils and are relatively safe for humans.

For future studies, a more extensive library of natural compounds must be scanned against SARS-CoV-2 variants’ RBDs using the proposed approach. Considering high-quality homology modeling results, the proposed approach could be used to build new models for emerging variants, such as new clades of Omicron (e.g., 21K, 21L, 22A, 22B, 22C, and 22D).

## Figures and Tables

**Table 1:** Interaction between RBDs and ACE2 as a result of MD trajectory clustering.

Variant	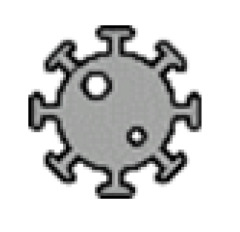	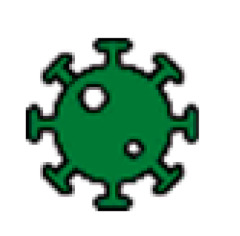	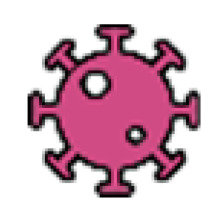	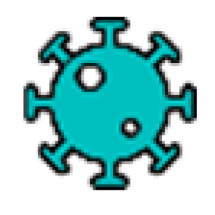	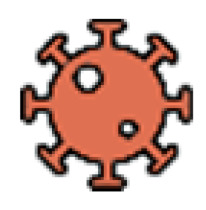	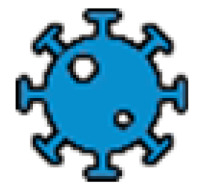	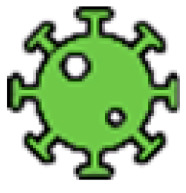	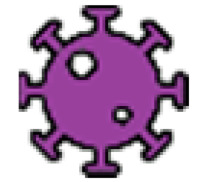	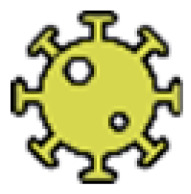
	Wild Type	Alpha	Beta	Gamma	Delta	Epsilon	Lambda	Mu	Omicron
R403					H34				
					HB ^1^				
K417	D30				D30	D30	D30		
	HB + SB				HB + SB	HB + SB + vdW	HB + SB		
Y449	D38	D38				D38		D38	
	HB + vdW	HB				HB		HB	
Y453			H34			H34			H34
			HB			vdW			π + vdW
L455				H34					
				vdW					
F456			T27						
			vdW						
K458				E23					
				HB + SB					
Y473		T27		E23				T27	
		HB		HB				vdW	
A475	S19	Q24		S19 + Q24		S19			
	HB	HB		2 HB		HB			
E484								E75	
								HB + SB	
F486				Q24					
				HB					
N487	Q24 + Y83	Y83	Y83	Y83	Y83	Q24 + Y83	Y83	Q24 + Y83	
	2 HB	HB	HB	HB	HB	2 HB	HB	2 HB	
Y489	Y83	Y83		Y83	Y83	Y83		Y83	
	HB	HB		HB	HB	HB		HB	
L492					K31			K31	
					HB			HB	
Q493		E35	K31	H34 + D38	K31 + E35	E35	E35	K31 + E35	E35
		HB	HB	2 HB	2 HB + vdW	HB	HB	2 HB	HB
G496	K353								
	HB + vdW								
Q498	Q42 + K353								
	3 HB								
T500	Y41		Y41 + N330	Y41 + N330		Y41 + N330	D355		D355
	HB		2 HB	2vdW + HB		2 HB	HB		HB
N501		Y41 + Q42	Y41						K353
		HB + π	π						HB
G502	K353		K353	K353		K353	K353		K353
	HB		HB	HB		HB	HB		HB
Y505			E37 + R393	E37					
			2 HB	HB					

^1^ HB—hydrogen bond; SB—salt bridge; vdW—Van der Waals interactions; π—π-π stacking.

**Table 2:** Ligands, TCM type, and their PubChem identifiers.

#	Ligand	TCM Type ^1^	PubChem CID	Molecular Formula	#	Ligand	TCM Type	PubChem CID	Molecular Formula
1	(−)-taxifolin	QPD	712316	C15H12O7	25	Glycyrrhizic acid	QPD, MSD, CP, GC	14982	C42H62O16
2	(+)-Epicatechin	QPD	182232	C15H14O6	26	Hederagenin	HPD	73299	C30H48O4
3	(2S)-dihydrobaicalein	QPD	14135323	C15H12O5	27	Herbacetin	MSD	5280544	C15H10O7
4	3-O-Methylviolanone	QPD	10019512	C18H18O6	28	Hesperidin	QPD, MSD	10621	C28H34O15
5	7-Methoxy-2-methyl isoflavone	DYY	354368	C17H14O3	29	Inflacoumarin A	MSD	5318437	C20H18O4
6	Amygdalin	QPD, MSD	656516	C20H27NO11	30	Isolicoflavonol	RDS	5318585	C20H18O6
7	Arenobufagin	LC	12305198	C24H32O6	31	Isotrifoliol	MSD	5318679	C16H10O6
8	Astragalus polysaccharide	HPD	2782115	C10H7ClN2O2S	32	Kaempferol	MSD, CP, DYY	5280863	C15H10O6
9	Baicalin	QPD, MSD	64982	C21H18O11	33	Kanzonol F	MSD	101666840	C26H28O5
10	Bufotalin	LC	12302120	C26H36O6	34	Licoisoflavone B	RDS	5481234	C20H16O6
11	Cianidanol	QPD	9064	C15H14O6	35	Luteolin	RDS	5280445	C15H10O6
12	Cinobufotalin	LC	259776	C26H34O7	36	Mairin	HPD	64971	C30H48O3
13	Cyclo(L-Tyr-l-Phe)	QPD	11438306	C18H18N2O3	37	naringenin	DYY, QPD	932	C15H12O5
14	Dammaradienyl acetate	HPD	179610	C32H52O2	38	Narirutin	QPD, MSD	442431	C27H32O14
15	Delphinidin	MSD	68245	C15H11ClO7	39	Neohesperidin	QPD, MSD	442439	C28H34O15
16	Desacetylcinobufotalin	LC	15513544	C24H32O6	40	Oxysanguinarine	HPD and TP	443716	C20H13NO5
17	Ephedrine	QPD, MSD	9294	C10H15NO	41	Quercetin	MSD, RDS, CP, DYY	5280343	C15H10O7
18	Eriodyctiol (flavanone)	QPD	373261	C15H12O6	42	Resivit	MSD	71629	C15H14O7
19	Estrone	MSD	5870	C18H22O2	43	Semilicoisoflavone-B	RDS	5481948	C20H16O6
20	Fisetin	RDS	5281614	C15H10O6	44	Sitosterol	MSD	222284	C29H50O
21	Formononetin	MSD, DYY	5280378	C16H12O4	45	SR-01000767148	QPD	676152	C16H14O6
22	Gamabufotalin	LC	259803	C24H34O5	46	Stigmasterol	MSD, HPD	5280794	C29H48O
23	Glyasperin F	RDS	392442	C20H18O6	47	telocinobufagin	LC	259991	C24H34O5
24	Glycyrrhetinic Acid	GC	10114	C30H46O4	48	ZINC13130930	QPD	25721350	C16H14O5

^1^ TCM (Tradicional Chinese Medicine), QPD (Qingfei Paidu Decoction), DYY (Da Yuan yin), MSD (Maxing Shigan Decoction), LC (Liushen Capsule), HPD and TP (Hubei Province Diagnosis and Treatment Protocol for COVID-19), RDS (Respiratory Detox Shot), GC (Gan cao), CP (96,606 classic prescriptions).

## Data Availability

Not applicable.

## Supplementary Materials

The following supporting information can be downloaded at: https://www.mdpi.com/article/10.3390/molecules27217336/s1, Table S1: Free binding energies of ligand-protein complexes (ΔG, kcal/mol); Table S2: Ligands’ SMILES codes.
