# Supplementary material for: Homology Modeling and Molecular Dynamics-Driven Search for Natural Inhibitors That Universally Target Receptor-Binding Domain of Spike Glycoprotein in SARS-CoV-2 Variants
Source: Molecules. 2022 Oct 28;27(21):7336. doi: 10.3390/molecules27217336 (PMC9657887; doi:10.3390/molecules27217336)
Supplement: Supplementary file 1 [file molecules-27-07336-s001.zip › molecules-1934062-supplementary.pdf]

Supporting information

Homology Modelling and Molecular Dynamics-Driven Search for Natural Inhibitors That Universally Target Receptor-Binding Domain of Spike Glycoprotein in SARS-Cov-2 Variants

Olha Ovchynnykova [1], Karina Kapusta [2,*], Natalia Sizochenko [3], Kostyantyn M. Sukhyy [1], Wojciech Kolodziejczyk [4], Glake A. Hill [4], and Julia Saloni [4]

1 Ukrainian State University of Chemical Technology, Dnipro, 49005, Ukraine;
2 Tougaloo College, Department of Chemistry and Physics, Tougaloo, MS, 39174, USA;
3 The Ronin Institute for Independent Scholarship, Montclair, NJ, 07043, USA;
4 Interdisciplinary Center for Nanotoxicity, Department of Chemistry, Physics and Atmospheric Sciences, Jackson State University, Jackson, MS, 39217, USA
* Correspondence: karina.kapusta@icnanotox.org

Table S1. Ligands' SMILES codes.

#	Compound	IUPAC Name	Canonical SMILES
1	(-)-taxifolin	(2S,3S)-2-(3,4-dihydroxyphenyl)-3,5,7-trihydroxy-2,3-dihydrochromen-4-one	C1=CC(=C(C=C1C2C(C(=O)C3=C(C=C(C=C3O2)O)O)O)O)O
2	(+)-Epicatechin	(2S,3S)-2-(3,4-dihydroxyphenyl)-3,4-dihydro-2H-chromene-3,5,7-triol	C1C(C(OC2=CC(=CC(=C21)O)O)C3=CC(=C(C=C3)O)O)O
3	(2S)-dihydrobaicalein	(2S)-5,6,7-trihydroxy-2-phenyl-2,3-dihydrochromen-4-one	C1C(OC2=C(C1=O)C(=C(C(=C2)O)O)O)C3=CC=CC=C3
4	3-O-Methylviolanone	(3S)-7-hydroxy-3-(2,3,4-trimethoxyphenyl)-2,3-dihydrochromen-4-one	COC1=C(C(=C(C=C1)C2COC3=C(C2=O)C=CC(=C3)O)OC)OC
5	7-Methoxy-2-methyl isoflavone	7-methoxy-2-methyl-3-phenylchromen-4-one	CC1=C(C(=O)C2=C(O1)C=C(C=C2)OC)C3=CC=CC=C3
6	Amygdalin	(2R)-2-phenyl-2-[(2R,3R,4S,5S,6R)-3,4,5-trihydroxy-6-[[(2R,3R,4S,5S,6R)-3,4,5-trihydroxy-6-(hydroxymethyl)oxan-2-yl]oxymethyl]oxan-2-yl]oxyacetonitrile	C1=CC=C(C=C1)C(C#N)OC2C(C(C(C(O2)COC3C(C(C(C(O3)CO)O)O)O)O)O)O
7	arenobufagin	5-[(3S,5R,8R,9S,10S,11S,13R,14S,17R)-3,11,14-trihydroxy-10,13-dimethyl-12-oxo-2,3,4,5,6,7,8,9,11,15,16,17-dodecahydro-1H-cyclopenta[a]phenanthren-17-yl]pyran-2-one	CC12CCC(CC1CCC3C2C(C(=O)C4(C3(CCC4C5=COC(=O)C=C5)O)C)O)O
8	Astragalus polysaccharide	2-(chloromethyl)-4-(4-nitrophenyl)-1,3-thiazole	C1=CC(=CC=C1C2=CSC(=N2)CCl)[N+](=O)[O-]
9	Baicalin	(2S,3S,4S,5R,6S)-6-(5,6-dihydroxy-4-oxo-2-phenylchromen-7-yl)oxy-3,4,5-trihydroxyoxane-2-carboxylic acid	C1=CC=C(C=C1)C2=CC(=O)C3=C(C(=C(C=C3O2)OC4C(C(C(C(O4)C(=O)O)O)O)O)O)O
10	bufotalin	[(3S,5R,8R,9S,10S,13R,14S,16S,17R)-3,14-dihydroxy-10,13-dimethyl-17-(6-oxopyran-3-yl)-1,2,3,4,5,6,7,8,9,11,12,15,16,17-tetradecahydrocyclopenta[a]phenanthren-16-yl] acetate	CC(=O)OC1CC2(C3CCC4CC(CCC4(C3CCC2(C1C5=COC(=O)C=C5)C)C)O)O
11	Cianidanol	(2R,3S)-2-(3,4-dihydroxyphenyl)-3,4-dihydro-2H-chromene-3,5,7-triol	C1C(C(OC2=CC(=CC(=C21)O)O)C3=CC(=C(C=C3)O)O)O
12	cinobufotalin	[(1R,2S,4R,5R,6R,7R,10S,11R,14S,16S)-14,16-dihydroxy-7,11-dimethyl-6-(6-oxopyran-3-yl)-3-oxapentacyclo[8.8.0.02,4.02,7.011,16]octadecan-5-yl] acetate	CC(=O)OC1C(C2(CCC3C(C24C1O4)CCC5(C3(CCC(C5)O)C)O)C)C6=COC(=O)C=C6
13	cyclo(L-Tyr-l-Phe)	(3S,6S)-3-benzyl-6-[(4-hydroxyphenyl)methyl]piperazine-2,5-dione	C1=CC=C(C=C1)CC2C(=O)NC(C(=O)N2)CC3=CC=C(C=C3)O
14	Dammaradienyl acetate	[(3S,8R,9R,10R,13R,14S,17S)-4,4,8,10,14-pentamethyl-17-(6-methylhepta-1,5-dien-2-yl)-2,3,5,6,7,9,11,12,13,15,16,17-dodecahydro-1H-cyclopenta[a]phenanthren-3-yl] acetate	CC(=CCCC(=C)C1CCC2(C1CCC3C2(CCC4C3(CCC(C4(C)C)OC(=O)C)C)C)C)C
15	Delphinidin	2-(3,4,5-trihydroxyphenyl)chromenylium-3,5,7-triol;chloride	C1=C(C=C(C(=C1O)O)O)C2=[O+]C3=CC(=CC(=C3C=C2O)O)O.[Cl-]
16	desacetylcinobufotalin	5-[(1R,2S,4R,5R,6R,7R,10S,11R,14S,16S)-5,14,16-trihydroxy-7,11-dimethyl-3-oxapentacyclo[8.8.0.02,4.02,7.011,16]octadecan-6-yl]pyran-2-one	CC12CCC(CC1(CCC3C2CCC4(C35C(O5)C(C4C6=COC(=O)C=C6)O)C)O)O
17	Ephedrine	(1R,2S)-2-(methylamino)-1-phenylpropan-1-ol	CC(C(C1=CC=CC=C1)O)NC

18	Eriodyctiol (flavanone)	(2R)-2-(3,4-dihydroxyphenyl)-5,7-dihydroxy-2,3-dihydrochromen-4-one	C1C(OC2=CC(=CC(=C2C1=O)O)O)C3=CC(=C(C=C3)O)O
19	Estrone	(8R,9S,13S,14S)-3-hydroxy-13-methyl-7,8,9,11,12,14,15,16-octahydro-6H-cyclopenta[a]phenanthren-17-one	CC12CCC3C(C1CCC2=O)CCC4=C3C=CC(=C4)O
20	Fisetin	2-(3,4-dihydroxyphenyl)-3,7-dihydroxychromen-4-one	C1=CC(=C(C=C1C2=C(C(=O)C3=C(O2)C=C(C=C3)O)O)O)O
21	Formononetin	7-hydroxy-3-(4-methoxyphenyl)chromen-4-one	COC1=CC=C(C=C1)C2=COC3=C(C2=O)C=CC(=C3)O
22	Gamabufotalin	5-[(3S,5R,8R,9S,10S,11R,13R,14S,17R)-3,11,14-trihydroxy-10,13-dimethyl-1,2,3,4,5,6,7,8,9,11,12,15,16,17-tetradecahydrocyclopenta[a]phenanthren-17-yl]pyran-2-one	CC12CCC(CC1CCC3C2C(CC4(C3(CCC4C5=COC(=O)C=C5)O)C)O)O
23	Glyasperin F	5,7-dihydroxy-3-(5-hydroxy-2,2-dimethylchromen-8-yl)-2,3-dihydrochromen-4-one	CC1(C=CC2=C(C=CC(=C2O1)C3COC4=CC(=CC(=C4C3=O)O)O)O)C
24	Glycyrrhetinic Acid	(2S,4aS,6aR,6aS,6bR,8aR,10S,12aS,14bR)-10-hydroxy-2,4a,6a,6b,9,9,12a-heptamethyl-13-oxo-3,4,5,6,6a,7,8,8a,10,11,12,14b-dodecahydro-1H-picene-2-carboxylic acid	CC1(C2CCC3(C(C2(CCC1O)C)C(=O)C=C4C3(CCC5(C4CC(CC5)(C)C(=O)O)C)C)C)C
25	Glycyrrhizic acid	(2S,3S,4S,5R,6R)-6-[(2S,3R,4S,5S,6S)-2-[[(3S,4aR,6aR,6bS,8aS,11S,12aR,14aR,14bS)-11-carboxy-4,4,6a,6b,8a,11,14b-heptamethyl-14-oxo-2,3,4a,5,6,7,8,9,10,12,12a,14a-dodecahydro-1H-picen-3-yl]oxy]-6-carboxy-4,5-dihydroxyoxan-3-yl]oxy-3,4,5-trihydroxyoxane-2-carboxylic acid	CC1(C2CCC3(C(C2(CCC1OC4C(C(C(C(O4)C(=O)O)O)O)OC5C(C(C(C(O5)C(=O)O)O)O)O)C)C(=O)C=C6C3(CCC7(C6CC(CC7)(C)C(=O)O)C)C)C)C
26	Hederagenin	(4aS,6aR,6aS,6bR,8aR,9R,10S,12aR,14bS)-10-hydroxy-9-(hydroxymethyl)-2,2,6a,6b,9,12a-hexamethyl-1,3,4,5,6,6a,7,8,8a,10,11,12,13,14b-tetradecahydropicene-4a-carboxylic acid	CC1(CCC2(CCC3(C(=CCC4C3(CCC5C4(CCC(C5(C)CO)O)C)C)C2C1)C)C(=O)O)C
27	Herbacetin	3,5,7,8-tetrahydroxy-2-(4-hydroxyphenyl)chromen-4-one	C1=CC(=CC=C1C2=C(C(=O)C3=C(O2)C(=C(C=C3O)O)O)O)O
28	Hesperidin	(2S)-5-hydroxy-2-(3-hydroxy-4-methoxyphenyl)-7-[(2S,3R,4S,5S,6R)-3,4,5-trihydroxy-6-[[(2R,3R,4R,5R,6S)-3,4,5-trihydroxy-6-methyloxan-2-yl]oxymethyl]oxan-2-yl]oxy-2,3-dihydrochromen-4-one	CC1C(C(C(C(O1)OCC2C(C(C(C(O2)OC3=CC(=C4C(=O)CC(OC4=C3)C5=CC(=C(C=C5)OC)O)O)O)O)O)O)O)O
29	Inflacoumarin A	7-hydroxy-4-(4-hydroxyphenyl)-6-(3-methylbut-2-enyl)chromen-2-one	CC(=CCC1=CC2=C(C=C1O)OC(=O)C=C2C3=CC=C(C=C3)O)C
30	Isolicoflavonol	3,5,7-trihydroxy-2-[4-hydroxy-3-(3-methylbut-2-enyl)phenyl]chromen-4-one	CC(=CCC1=C(C=CC(=C1)C2=C(C(=O)C3=C(C=C(C=C3O2)O)O)O)O)C
31	Isotrifoliol	3,9-dihydroxy-1-methoxy-[1]benzofuro[3,2-c]chromen-6-one	COC1=CC(=CC2=C1C3=C(C4=C(O3)C=C(C=C4)O)C(=O)O2)O
32	Kaempferol	3,5,7-trihydroxy-2-(4-hydroxyphenyl)chromen-4-one	C1=CC(=CC=C1C2=C(C(=O)C3=C(C=C(C=C3O2)O)O)O)O
33	Kanzonol F	(1R,13R)-15-methoxy-7,7-dimethyl-16-(3-methylbut-2-enyl)-8,12,20-trioxapentacyclo[11.8.0.02,11.04,9.014,19]henicosa-2(11),3,5,9,14(19),15,17-heptaen-17-ol	CC(=CCC1=C(C2=C(C=C1O)OCC3C2OC4=C3C=C5C=CC(OC5=C4)(C)C)OC)C
34	Licoisoflavone B	5,7-dihydroxy-3-(5-hydroxy-2,2-dimethylchromen-6-yl)chromen-4-one	CC1(C=CC2=C(O1)C=CC(=C2O)C3=COC4=CC(=CC(=C4C3=O)O)O)C
35	Luteolin	2-(3,4-dihydroxyphenyl)-5,7-dihydroxychromen-4-one	C1=CC(=C(C=C1C2=CC(=O)C3=C(C=C(C=C3O2)O)O)O)O
36	Mairin	(1R,3aS,5aR,5bR,7aR,9S,11aR,11bR,13aR,13bR)-9-hydroxy-5a,5b,8,8,11a-pentamethyl-1-prop-1-en-2-yl-1,2,3,4,5,6,7,7a,9,10,11,11b,12,13,13a,13b-hexadecahydrocyclopenta[a]chrysene-3a-carboxylic acid	CC(=C)C1CCC2(C1C3CCC4C5(CCC(C(C5CCC4(C3(CC2)C)C)(C)C)O)C)C(=O)O
37	naringenin	5,7-dihydroxy-2-(4-hydroxyphenyl)-2,3-dihydrochromen-4-one	C1C(OC2=CC(=CC(=C2C1=O)O)O)C3=CC=C(C=C3)O
38	Narirutin	(2S)-5-hydroxy-2-(4-hydroxyphenyl)-7-[(2S,3R,4S,5S,6R)-3,4,5-trihydroxy-6-[[(2R,3R,4R,5R,6S)-3,4,5-trihydroxy-6-methyloxan-2-yl]oxymethyl]oxan-2-yl]oxy-2,3-dihydrochromen-4-one	CC1C(C(C(C(O1)OCC2C(C(C(C(O2)OC3=CC(=C4C(=O)CC(OC4=C3)C5=CC=C(C=C5)O)O)O)O)O)O)O)O
39	Neohesperidin	(2S)-7-[(2S,3R,4S,5S,6R)-4,5-dihydroxy-6-(hydroxymethyl)-3-[(2S,3R,4R,5R,6S)-3,4,5-trihydroxy-6-methyloxan-2-yl]oxyoxan-2-yl]oxy-5-hydroxy-2-(3-hydroxy-4-methoxyphenyl)-2,3-dihydrochromen-4-one	CC1C(C(C(C(O1)OC2C(C(C(OC2OC3=CC(=C4C(=O)CC(OC4=C3)C5=CC(=C(C=C5)OC)O)O)CO)O)O)O)O)O
40	Oxysanguinarine	24-methyl-5,7,18,20-tetraoxa-24-azahexacyclo[11.11.0.02,10.04,8.014,22.017,21]tetracosa-1(13),2,4(8),9,11,14(22),15,17(21)-octaen-23-one	CN1C2=C(C=CC3=CC4=C(C=C32)OCO4)C5=C(C1=O)C6=C(C=C5)OCO6
41	Quercetin	2-(3,4-dihydroxyphenyl)-3,5,7-trihydroxychromen-4-one	C1=CC(=C(C=C1C2=C(C(=O)C3=C(C=C(C=C3O2)O)O)O)O)O
42	Resivit	2-(3,4-dihydroxyphenyl)-3,4-dihydro-2H-chromene-3,4,5,7-tetrol	C1=CC(=C(C=C1C2C(C(C3=C(C=C(C=C3O2)O)O)O)O)O)O
43	Semilicoisoflavone-B	5,7-dihydroxy-3-(8-hydroxy-2,2-dimethylchromen-6-yl)chromen-4-one	CC1(C=CC2=C(O1)C(=CC(=C2)C3=COC4=CC(=CC(=C4C3=O)O)O)O)C
44	Sitosterol	(3S,8S,9S,10R,13R,14S,17R)-17-[(2R,5R)-5-ethyl-6-methylheptan-2-yl]-10,13-dimethyl-2,3,4,7,8,9,11,12,14,15,16,17-dodecahydro-1H-cyclopenta[a]phenanthren-3-ol	CCC(CCC(C)C1CCC2C1(CCC3C2CC=C4C3(CCC(C4)O)C)C)C(C)C
45	SR-01000767148	(2R)-5,7-dihydroxy-2-(3-hydroxy-4-methoxyphenyl)-2,3-dihydrochromen-4-one	COC1=C(C=C(C=C1)C2CC(=O)C3=C(C=C(C=C3O2)O)O)O
46	Stigmasterol	(3S,8S,9S,10R,13R,14S,17R)-17-[(E,2R,5S)-5-ethyl-6-methylhept-3-en-2-yl]-10,13-dimethyl-2,3,4,7,8,9,11,12,14,15,16,17-dodecahydro-1H-cyclopenta[a]phenanthren-3-ol	CCC(C=CC(C)C1CCC2C1(CCC3C2CC=C4C3(CCC(C4)O)C)C)C(C)C
47	telocinobufagin	5-[(3S,5S,8R,9S,10R,13R,14S,17R)-3,5,14-trihydroxy-10,13-dimethyl-2,3,4,6,7,8,9,11,12,15,16,17-dodecahydro-1H-cyclopenta[a]phenanthren-17-yl]pyran-2-one	CC12CCC(CC1(CCC3C2CCC4(C3(CCC4C5=COC(=O)C=C5)O)C)O)O
48	ZINC13130930	(2R)-5,7-dihydroxy-6-methoxy-2-phenyl-2,3-dihydrochromen-4-one	COC1=C(C2=C(C=C1O)OC(CC2=O)C3=CC=CC=C3)O

Table S2. Free binding energies of ligand-protein complexes (ΔG, kcal/mol)

#	ACE2	SARS-CoV	SARS-CoV-2								
			Wild	Alpha	Beta	Gamma	Delta	Epsilon	Lambda	Mu	Omicron
1	-24.0	-70.8	-49.1	-43.3	-26.1	-20.4	-35.6	-35.9	-34.2	-33.4	-32.57
2	-26.0	-36.7	-44.4	-43.0	-37.4	-24.4	-34.0	-36.7	-32.9	-36.1	-10.89
3	-27.4	-37.0	-48.8	-43.5	-37.5	-28.1	-41.3	-31.9	-29.6	-38.3	-46.78
4	-18.6	-33.4	-42.7	-40.9	-29.4	-43.5	-28.6	-25.0	-27.9	-32.7	-27.11
5	-19.9	-27.3	-48.3	-39.2	-29.7	-39.3	-32.7	-27.8	-21.0	-24.4	-34.11
6	-32.9	-49.3	-68.2	-53.7	-39.2	-29.2	-47.9	-43.3	-47.2	-52.0	-46.26
7	-46.8	-49.0	-43.0	-50.2	-39.3	-42.3	-37.5	-38.3	-32.9	-32.2	-41.42
8	-24.8	-25.9	-30.3	-20.6	-31.7	-22.9	-30.7	-35.4	-25.1	-30.4	-34.52
9	-28.3	-37.8	-50.4	-45.1	-38.1	-39.4	-45.3	-48.6	-35.9	-44.4	-51.64
10	N/A	-37.3	-34.7	N/A	-40.5	-50.3	-40.3	-37.9	-22.3	-31.7	-48.34
11	-22.1	-51.5	-55.2	-18.1	-46.8	-28.2	-32.5	-39.4	-37.2	-35.9	-26.44
12	-26.2	-31.5	-39.6	-46.8	-34.4	-26.1	-38.8	-38.6	-34.5	-42.8	-49.66
13	-25.8	-49.4	-53.6	-26.0	-38.8	-32.6	-45.2	-43.5	-26.1	-41.7	-47.84
14	N/A	-42.4	-44.1	-16.7	-40.0	N/A	-46.7	N/A	-27.7	N/A	-46.35
15	-36.8	-75.8	-46.7	-27.8	-44.2	-77.7	-39.1	-40.6	-32.6	-36.4	-25.34
16	-24.5	-38.3	-45.9	-50.7	-39.4	-33.0	-32.8	-48.3	-31.0	-42.9	-46.28
17	N/A	N/A	-35.1	-31.4	-31.3	-10.5	-16.3	-34.0	-25.0	-26.9	-25.48
18	-16.7	-40.1	-55.5	-9.4	-27.6	-25.5	-36.4	-38.4	-29.0	-33.0	-26.73
19	-30.2	-37.6	-47.3	-26.6	-40.7	-27.6	-18.2	-37.3	-27.3	-31.8	-34.52
20	-29.3	-67.3	-51.5	-38.4	-38.3	-34.5	-20.1	-37.0	-31.3	-39.5	-29.64
21	-30.8	-38.8	-35.5	-28.7	-36.3	-40.6	-37.8	-23.2	-29.8	-36.1	-35.44
22	-25.5	-45.4	-41.5	-42.9	-42.3	-27.9	-28.2	-44.0	-27.1	-40.0	-39.65
23	-30.8	-43.1	-48.4	-43.1	-34.9	-32.4	-33.7	-29.9	-24.8	-37.4	-37.68
24	-20.8	-36.1	-43.8	-24.2	-34.3	-28.5	-45.0	-33.1	-23.5	-44.3	-38.53
25	-41.8	-49.2	-62.7	-42.0	-50.8	-29.4	-37.9	-51.3	-48.7	-43.1	-36.18
26	-24.3	-30.0	-34.6	-3.9	-29.3	-48.9	-37.6	-31.5	-19.7	-43.1	-35.12
27	-31.7	-41.4	-48.9	-21.0	-37.7	-27.4	-27.1	-32.3	-30.6	-36.6	-40.26
28	-37.4	-58.1	-63.9	-50.1	-73.7	-30.8	-39.1	-61.4	-50.4	-65.2	-69.29
29	-29.0	-46.1	-74.4	-29.7	-44.1	-29.3	-49.3	-43.5	-41.5	-44.2	-50.31
30	-24.1	-47.8	-51.7	-41.8	-31.2	-34.4	-38.0	-33.8	-34.1	-42.5	-46.87
31	-19.5	-40.6	-42.6	-29.2	-35.5	-20.0	-22.4	-22.5	-27.5	-35.2	-23.55
32	-22.2	-41.6	-50.4	-26.3	-20.1	-19.4	-23.8	-32.1	-28.4	-40.1	-34.39
33	-23.7	-48.4	-61.4	-34.4	-44.7	-78.4	-40.9	-44.8	-29.6	-50.0	-46.93
34	-23.6	-37.8	-42.9	-13.1	-41.2	-9.7	-22.9	-27.4	-24.1	-34.5	-41.84
35	-26.7	-37.1	-40.0	-30.5	-38.6	-26.4	-24.1	-45.7	-32.5	-26.0	-29.02
36	-4.5	-32.2	-34.0	-1.0	-31.9	-20.6	-24.5	-55.9	-22.7	-33.2	-46.49
37	-19.4	-28.3	-48.6	-26.9	-29.3	-30.2	-28.5	-28.9	-29.8	-33.6	-24.69
38	-44.8	-42.7	-68.9	-72.1	-51.4	-84.3	-62.0	-67.7	-40.7	-51.8	-60.67
39	-42.6	-58.3	-67.4	-60.8	-76.0	-45.6	-40.9	-65.1	-49.7	-63.2	-65.7

40	-27.4	-73.0	-48.9	-34.4	-38.4	-39.0	-58.4	-36.4	-50.6	-41.6	-40.86
41	-29.7	-40.5	-44.1	-33.5	-44.2	-17.2	-38.1	-41.6	-31.7	-35.8	-35.19
42	-26.5	-48.6	-44.8	-41.5	-36.4	-24.6	-33.0	-31.4	-27.3	-31.7	-46.42
43	-35.7	-33.4	-35.5	-20.7	-25.2	-41.5	-41.5	-34.0	-28.7	-33.9	-22.14
44	-15.6	N/A	-47.1	-41.3	-38.4	-39.5	-35.0	-45.9	-21.7	-40.7	-39.14
45	-27.6	-42.0	-46.5	-35.4	-35.6	-22.9	-32.4	-36.7	-27.9	-35.9	-39.93
46	-11.9	-53.4	-51.8	-38.9	-31.8	-41.7	-46.9	-36.8	N/A	-47.7	-42.56
47	-47.2	-46.3	-39.0	-35.9	-36.9	-23.6	-45.1	-38.7	-26.3	-42.7	-38.83
48	-31.0	-36.1	-40.5	-32.4	-36.9	-26.1	-38.4	-38.4	-26.0	-35.9	-44.82
